# Morphological and physiological adaptation characteristics of lithophytic bryophytes to karst high calcium environment

**DOI:** 10.1186/s12870-022-03980-4

**Published:** 2023-03-25

**Authors:** Wenping Meng, Jingcheng Ran, Quanhou Dai, Na Tu, Tingjiao Leng, Qingqing Ren

**Affiliations:** 1 Guizhou Botanical Garden, Guiyang, 550001 China; 2 Guizhou Academy of Forestry Sciences, Guiyang, 550005 China; 3 College of Forestry, Guizhou Universtry, Guiyang, 550001 China; 4 Institute for Forest Resources & Environment of Guizhou, Guiyang, 550001 China; 5 The Second Affiliated Hospital of Zunyi Medical University, Zunyi, 563000 China

**Keywords:** Karst, High calcium environment, Lithophytic bryophyte, Physiological regulation, Adaptation mechanism

## Abstract

**Background:**

Lithophytic bryophytes grow on the rock surface, change the habitat on the rock surface through biological karstification, and provide a material basis for the growth of other plants. However, the surface calcium content of bare rock is high. The lithophytic bryophytes may have a special mechanism to adapt to the karst high calcium environment. The present study aimed to explore the physiological regulation process of karst lithophytic bryophytes under high calcium environment, and to provide scientific basis for revealing the maintenance mechanism of karst biodiversity.

**Results:**

With the increase of Ca^2+^ concentration, the contents of Pro, SP and MDA of lithophytic bryophytes showed a downward—upward—downward trend. However, when Ca^2+^ ≥ 400 mmol/L, the contents of Pro and SP changed significantly at 1d, 2d, 3d, 5d and 7d with the extension of culture time, and lithophytic bryophytes died after 2 months of culture. Under different Ca^2+^ concentrations, the maximum SOD activity of lithophytic bryophytes is 1758.00 (U/g FW), the minimum is 92.60 (U/g FW), the maximum POD activity is 120.88 (U/g FW), and the minimum is 4.80 (U/g FW). The antioxidative activity of of *Hyophila involuta* are higher than that of *Didymodon constrictus* and *Eurohypnum leptothallum*, and its enzyme activity changed significantly with the increase of calcium concentration and time.At the same time, the contents of TChl, Chla, and Chlb in lithophytic bryophytes decreased with the increase of Ca^2+^ concentration. When Ca^2+^ = 400 mmol/L, the contents of TChl and Chla were the lowest, but when Ca^2+^ > 400 mmol/L, they began to increase. In addition, ABA is negatively correlated with TChl and Chla, and positively correlated with ROS. It shows that ABA has a certain role in regulating the adaptation of lithophytic bryophytes to high calcium environment.

**Conclusions:**

Lithophytic bryophytes have strong calcium tolerance, and their physiological response to high calcium stress is different from vascular bundle plants. The general stress principle is not applicable to lithophytic bryophytes. The response of lithophytic bryophytes to the change of Ca^2+^concentration is slow, showing passive response or inert response.

## Background

Karst landforms are widely distributed in the world, accounting for about 15% of the global land area [1]. The karst in Southwest China centered on Guizhou covers an area of more than 510,000 km^2^, which is the largest and most concentrated karst landform in the world [2]. Karst landforms are mainly formed by carbonate rocks (CaCO_3_, MgCO_3_), which are characterized by shallow soil layers, poor water holding capacity, and high soil calcium content [3, 4]. The high content of Ca in soil is one of the most important environmental factors affecting its vegetation type and distribution, as well as plant physiological characteristics. The concentration of Ca^2+^ in root, stem, leave, cell wall, vacuole and other organizational structure of plant growing on karst calcareous soil is significantly higher than that in non karst areas [5–7]. For example, the content of Ca in the leaves of common calcareous plants in Maolan karst forest is higher than 10 g/kg, which is five times the content of Ca required by terrestrial higher plants [8]. Different plant types have different ability to absorb and enrich Ca. For example, the average Ca content in karst lithophytic bryophyte is 1.903% [9], in fern is 1.25%, in shrub and herb plants is 1.909% [10], and in arbor is 2.16% [11]. Plants in karst areas have high calcium content, but they can still grow normally, indicating that these plants have special mechanisms in physiological regulation.Previous studies have shown that under natural growth conditions, the SOD activity, POD activity, SP content and Pro content of calcium loving plants *Carpinus pubescens*, *Eurycorymbus cavaleriei*,*Kmeria septentrionalis*, and random plant *Quercus glauca* are higher than those of calcium intolerant plant *Pinus armandii*,*Camellia oleifera*) [12].Researcher found that under calcium stress, *Cornus wilsoniana* have higher CAT activity and SS content than *choerospondias axillaris*, which are suitable plants in karst high calcium environment [13]. By testing the stress resistance indexes of plants under different calcium environments, it was found that the changes of total calcium content in rhizosphere soil, proline content in leaves, POD and SOD activities of calcium loving plants respectively were w (Ca) = 32.44–43.98 g/kg, w (Pro) = 97.53–183.62 μg/g, w(POD)6.21–38.43μKat,w(SOD)1.47–2.48μKat,slightly higher than intermediate plants, is 1–2 orders of magnitude higher than calcium intolerant plants [14]. In order to reproduce and develop, plants in karst areas have gradually formed morphological structures that adapt to the living environment in the long-term evolution process [15]. The leaves of plants in karst areas are mostly xerophytic, such as leathery, waxy, downy, thick leaves, and obvious mesophyll differentiation [6, 7, 16].For example, the upper epidermis of *Lonicera* plants in karst areas has many groove like concave structures, which can slow down the rate of water exchange between plants and the outside world [17]. And there are small and dense lower epidermal pores (328 mm^2^) under dense epidermal hairs, which can inhibit transpiration rate and improve photosynthetic efficiency [17].*Loropetalum chinense* grown in karst areas and non karst areas has significant differences in leaf width and thickness, stomatal distribution frequency, upper epidermal cell size and palisade tissue thickness [18]. The upper and lower epidermal cells of *Loropetalum chinense* in karst areas tend to be smaller than those in non karst areas, the number of cells per unit area decreases [18]. The thickness of upper epidermal cells in karst areas increases, and their arrangement is staggered and inlaid with each other, while the shape of upper epidermal cells in non karst areas is relatively regular, mostly irregular quadrilateral [18]. Root system is the main organ for plants to absorb water and nutrients. Its biomass, morphology and distribution are affected by site conditions, soil layer thickness, water, nutrients, community structure and species composition, and characteristics [19, 20]. In karst areas, the proportion of fine root biomass to total root biomass of the same plant is less than that in sandy environment, and the root biomass in sandy environment is mainly vertically distributed [21, 22].

The research on the adaptation mechanism of karst plants to high calcium environment mainly focuses on vascular plants, while there is very little research on non vascular plants. Bryophytes are transitional plant types from aquatic to terrestrial, and have strong tolerance to extreme environments. As a pioneer plant in the process of ecosystem succession, bryophytes play an important role in promoting ecosystem nutrient utilization and circulation, water and soil conservation, and maintaining ecosystem structure and function [23].In recent years, the research of lithophytic bryophytes has made a lot of research results, mainly focusing on the flora, species diversity, population characteristics, pollutant monitoring, tolerance to exogenous calcium and the change characteristics of some physiological indicators [24–27].

However, there are still some shortcomings, such as (1) the relationship between the biodiversity of lithophytic bryophytes and their community composition and the environment in karst areas, (2) the functional traits of karst lithophytic bryophytes, (3) the regulation mechanism of lithophytic bryophytes on high calcium environment Wait.Therefore, in this study, the dominant species of karst lithophytic bryophytes were selected as the research objects, and the morphological changes and physiological regulation processes of karst lithophytic bryophytes were observed by culturing them in different concentrations of Ca^2+^ solutions at different times, and then to reveal the effect of lithophytic bryophytes on karst high Ca adaptation mechanisms to the environment. This study can enrich the scientific theory of plant physiology and ecology in karst areas, provide scientific theoretical support for the study of plant physiological evolution history and the maintenance mechanism of biodiversity in karst areas, and provide new ideas and methods for the ecological management of rocky desertification.

## Results

### Effect of high calcium stress on the growth of lithophytic Bryophytes

#### Effects of high calcium stress on the morphology of lithophytic Bryophytes

**Fig. 1:**
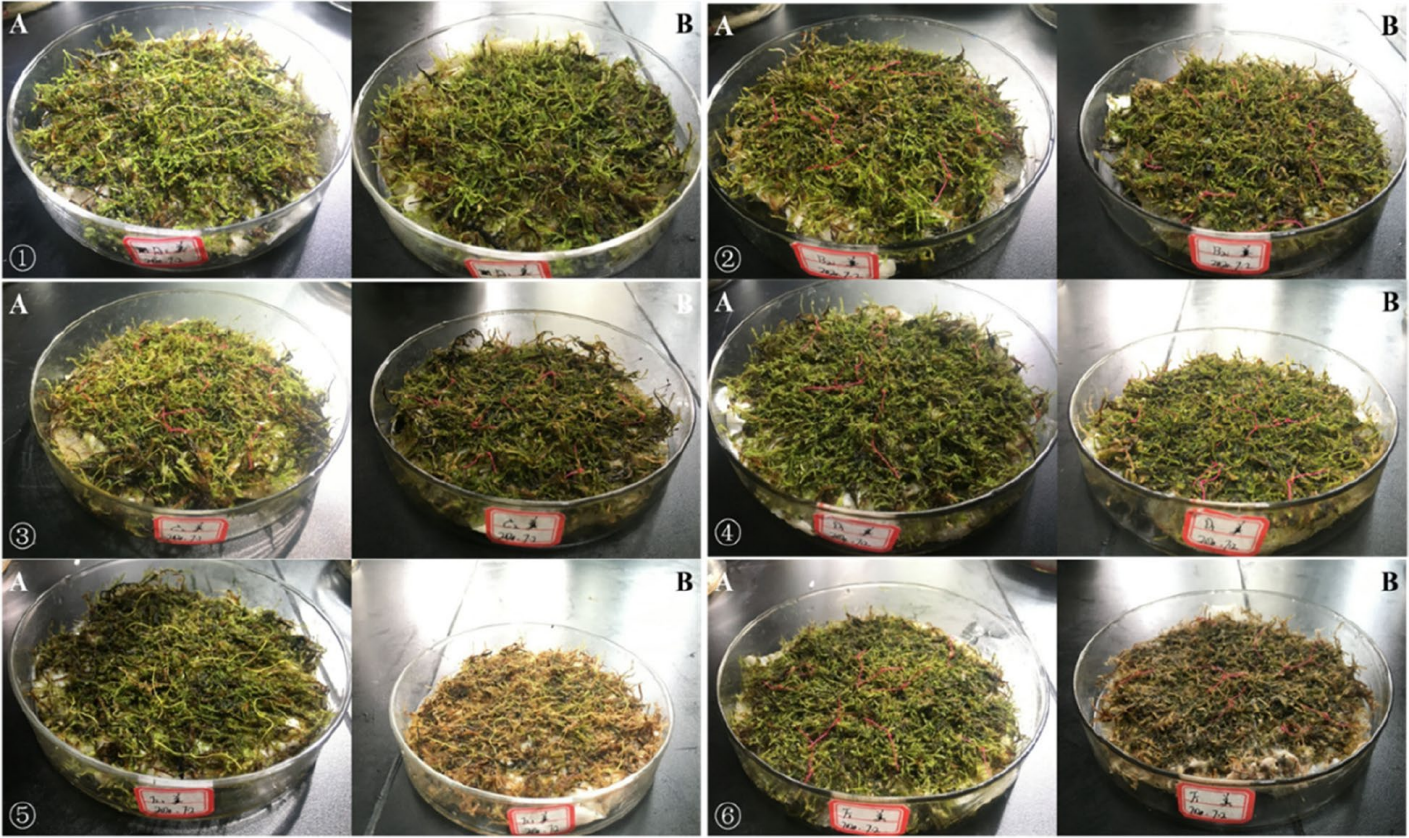
Growth of *Eurohypnum leptothallum* in different calcium environments. **A** the growth state of *Eurohypnum leptothallum* before treatment with Ca^2+^ solution, (**B**) the growth state of *Eurohypnum leptothallum* after 2 months of culture with Ca^2+^ solution.①:Ca^2+^ concentration is 0 mmol/L, ②:Ca^2+^ concentration is 50 mmol/L, ③: Ca^2+^concentration is 100 mmol/L, ④: Ca^2+^ concentration is 200 mmol/L, ⑤: Ca^2+^ concentration is 400 mmol/L, ⑥: Ca^2+^ concentration is 800 mmol/L

**Fig. 2:**
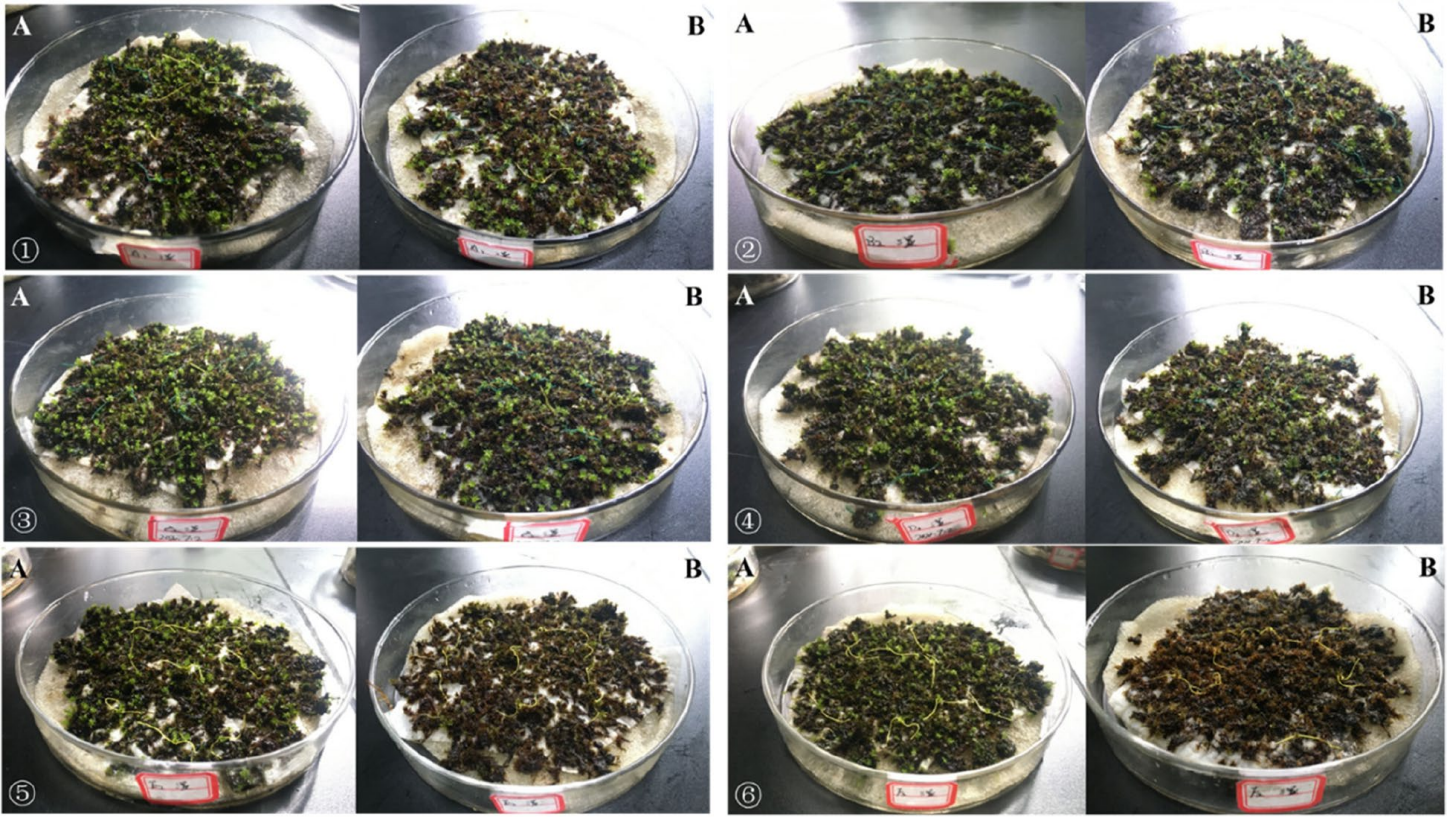
Growth of *Hyophila involute* in different calcium environments. **A** the growth state of *Hyophila involuta* before treatment with Ca^2+^ solution, (**B**) the growth state of *Hyophila involuta* after 2 months of culture with Ca^2+^ solution. ①:Ca^2+^ concentration is 0 mmol/L, ②:Ca^2+^ concentration is 50 mmol/L, ③:Ca^2+^ concentration is 100 mmol/L, ④:Ca^2+^ concentration is 200 mmol/L, ⑤:Ca^2+^ concentration is 400 mmol/L, ⑥:Ca^2+^ concentration is 800 mmol/L

**Fig. 3:**
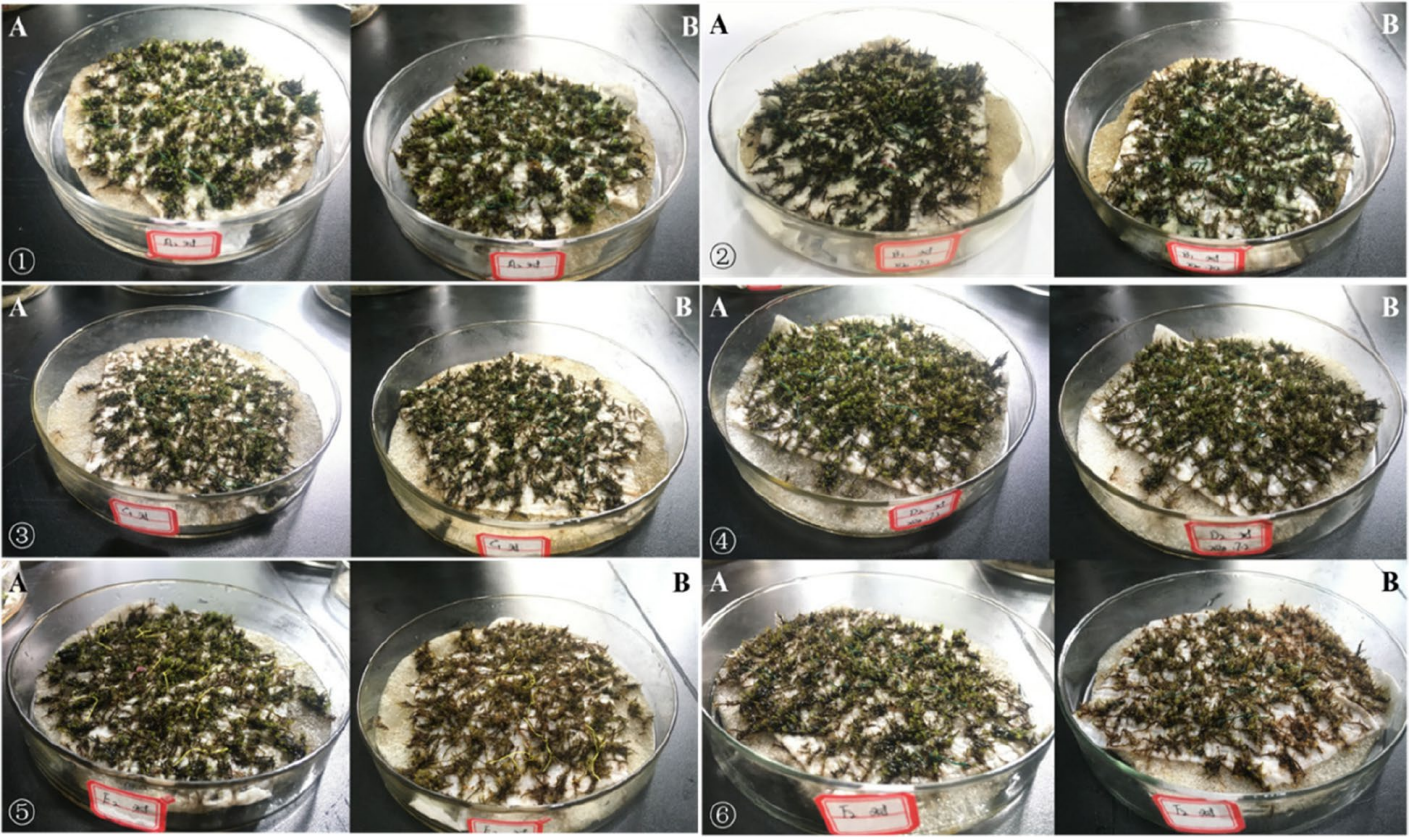
Growth of *Didymodon constrictus* in different calcium environments. **A** the growth state of *Didymodon constrictus* before treatment with Ca^2+^ solution, (**B**) the growth state of *Didymodon constrictu* after 2 months of culture with Ca^2+^ solution. ①:Ca^2+^concentration is 0 mmol/L, ②:Ca^2+^ concentration is 50 mmol/L, ③:Ca^2+^ concentration is 100 mmol/L, ④:Ca^2+^concentration is 200 mmol/L, ⑤:Ca^2+^ concentration is 400 mmol/L, ⑥: Ca^2+^concentration is 800 mmol/L

It can be seen from that *Eurohypnum leptothallum *(Fig. 1), *Hyophila involuta *(Fig. 2), *Didymodon constrictus *(Fig. 3),when the concentration of Ca^2+^ is 0 mmol/L, 50 mmol/L, 100 mmol/L and 200 mmol/L, the plant color is normal after two months of culture in different concentrations of Ca^2+^ solution; When the concentration of ca^2+^ was 400 mmol/L and 800 mmol/L, the plant changed from green to yellow and died.

### Growth of lithophytic bryophytes under high calcium stress

In the first month, *Didymodon constrictus*, *Eurohypnum leptothallum*, *Hyophila involuta* all grew in different Ca^2+^ solutions (Table 1), but when the concentration of Ca^2+^was between 50 mmol/L and 200 mmol/L, the growth of lithophytic bryophytes was more, and the growth of different species was different under different Ca^2+^ concentrations. *Eurohypnum leptothallum* increased more at 0 mmol/L, 100 mmol/L and 200 mmol/L. The growth of *Hyophila involuta* was the largest at 100 mmol/L and 200 mmol/L. The growth of *Didymodon constrictu* was larger at 50 mmol/L, 100 mmol/L and 200 mmol/L. In the second month, *Eurohypnum leptothallum* showed negative growth when Ca^2+^ = 800 mmol/L, *Didymodon constrictus* showed negative growth When Ca^2+^ ≥ 100 mmol/L, *Hyophila involuta* showed negative growthWhen Ca^2+^ ≥ 400 mmol/L. According to the growth of three lithophytic bryophytes under different Ca^2+^ concentrations, the suitable growth concentration of *Didymodon constrictus* is Ca^2+^ ≤ 100 mmol/L;Ca^2+^ ≤ 200 mmol/L is the suitable growth concentration for *Hyophila involute* and *Eurohypnum leptothallum*. The change of plant morphology can most intuitively reflect the impact of environmental stress on plants, but it lags behind the physiological response. Once it causes damage, it is difficult to recover.

**Table 1:** height growth of lithophyte bryophytes under different concentrations of Ca^2+^

Species Ca^2+^	*Eurohypnum leptothallum*			*Hyophila involuta*			*Didymodon constrictus*		
	Increase in first month(mm)	Increase in second month(mm)	Increase in 2 month(mm)	Increase in first month(mm)	Increase in second month(mm)	Increase in 2 month(mm)	Increase in first month(mm)	Increase in second month(mm)	Increase in 2 month(mm)
0 mmol/L	2.70	5.13	7.83	1.30	0.16	1.46	1.05	0.40	1.45
50 mmol/L	0.70	0.49	1.19	1.40	0.18	1.58	1.33	0.03	1.35
100 mmol/L	1.78	0.35	2.13	2.00	0.10	2.10	1.35	-0.09	1.26
200 mmol/L	2.30	0.27	2.57	2.00	0.24	2.24	1.33	-0.53	0.80
400 mmol/L	1.03	0.16	1.20	1.40	-0.10	1.30	0.75	-0.20	0.55
800 mmol/L	1.25	-0.45	0.80	1.05	-0.14	0.91	0.80	-0.43	0.37

### Change characteristics of osmotic adjustment substances

#### Variation characteristics of proline (Pro) content

**Fig. 4:**
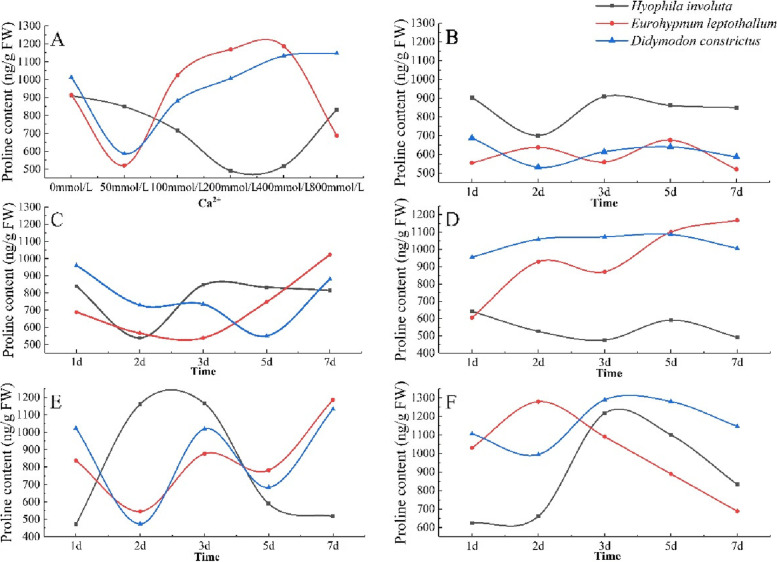
Changes of Pro in lithophytic bryophytes in different calcium environments. **A** The changes of Pro content of three lithophytic bryophytes cultured in different concentrations of Ca^2+^ solution for 7 days. **B** The change of Pro content of three lithophytic bryophytes after 7 days in Ca^2+^ 50 mmol/L solution. **C** The change of Pro content of three lithophytic bryophytes after 7 days in Ca^2+^ 100 mmol/L solution. **D** The change of Pro content of three lithophytic bryophytes after 7 days in Ca^2+^ 200 mmol/L solution. **E** The change of Pro content of three lithophytic bryophytes after 7 days in Ca^2+^ 400 mmol/L solution. **F** Changes in Pro content of three lithophytic bryophytes after 7 days of cultivation in Ca^2+^ 800 mmol/L solution

The Pro contents of the three lithophytic bryophytes showed a decreasing-rising-decreasing trend with the increase of Ca^2+^ concentration, but there were differences in the Pro contents and changing trends of different species under different Ca^2+^concentrations (Fig. 4-A).When Ca^2+^ = 50 mmol/L, the Pro content of *Eurohypnum leptothallum* and *Didymodon constrictus* is the lowest, when Ca^2+^ ≥ 100 mmol/L, the Pro content rises sharply, and when Ca^2+^ ≥ 400 mmol/L, the Pro content of both shows a steady downward trend.When Ca^2+^ = 200 mmol/L, Pro content of *Hyophila involute* is the lowest, when 200 mmol/L ≤ Ca^2+^ ≤ 400 mmol/L, when Ca^2+^ > 400 mmol/L Pro content increased sharply.

With the extension of culture time, the variation trend and range of Pro content in lithophytic bryophytes are different at the same concentration.When the concentration of Ca^2+^ = 50 mmol/L, the change trend of Pro content of *Hyophila involute* and *Didymodon constrictus* with the extension of culture time is the same, which decreases in 2 days and increases in 3 days, while the change trend of *Eurohypnum leptothallum* is opposite to the former two (Fig. 4-B).However, when Ca^2+^ = 100 mmol/L, the Pro content of *Eurohypnum leptothallum* decreased from 1 to 3 days, and began to rise after 3 days *Didymodon constrictus* decreased from 1 to 5 days, and began to rise after 5 days (Fig. 4-C).When Ca^2+^ = 200 mmol/L, the Pro content of *Eurohypnum leptothallum* and *Didymodon constrictus* was significantly higher than that of *Hyophila involute*, and the Pro content of *Eurohypnum leptothallum* increased with the extension of culture time. *Didymodon constrictus* was in a stable state, and *Hyophila involute* decreased in 1 days -3 days, increased in 3 days -5 days, and decreased after 5 days (Fig. 4-D).When Ca^2+^ = 400 mmol/L, the Pro content of *Eurohypnum leptothallum* and *Didymodon constrictus* showed a downward—upward – downward—upward trend with the extension of culture time, *Hyophila involute* increased first and then decreased (Fig. 4-E).When Ca^2+^ = 800 mmol/L, the Pro content of *Didymodon constrictus* decreased in 1 days -2 days and increased in 2 days -3 days, *Eurohypnum leptothallum* rose in 1 days -2 days, *Hyophila involute* increased in 1 days -2 days. And three lithophytic bryophytes showed a downward trend after 3 days (Fig. 4-F).

### Change characteristics of soluble protein (SP) content

**Fig. 5:**
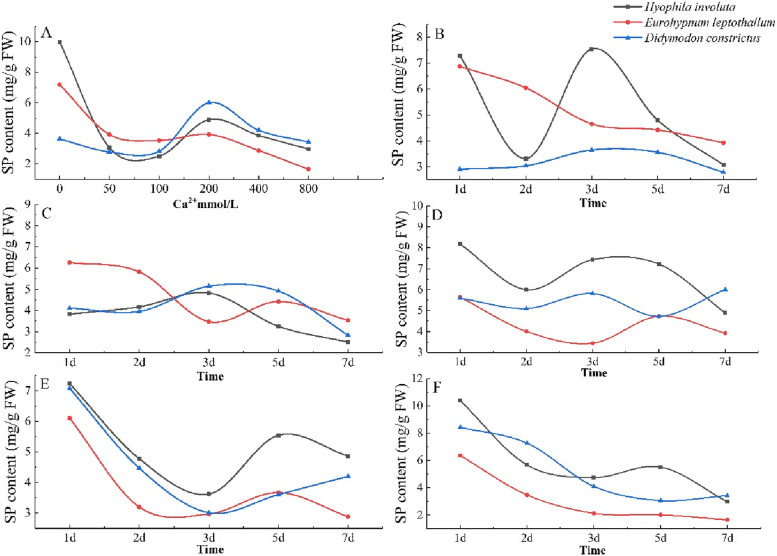
Changes of SP in lithophytic bryophytes in different calcium environments. **A** The changes of SP content of three lithophytic bryophytes cultured in different concentrations of Ca^2+^ solution for 7 days. **B** Changes in SP content of three lithophytic bryophytes after 7 days of culture in Ca^2+^ 50 mmol/L solution. **C** Changes of SP content of three lithophytic bryophytes after 7 days of culture in Ca^2+^ 100 mmol/L solution. **D** Changes in SP content of three lithophytic bryophytes after 7 days of culture in Ca^2+^ 200 mmol/L solution. **E** Changes of SP content of three lithophytic bryophytes after 7 days of culture in Ca^2+^ 400 mmol/L solution. **F** Changes in SP content of three lithophytic bryophytes after 7 days of culture in Ca^2+^ 800 mmol/L solution

The SP content in the lithophytic bryophytes showed a downward upward downward trend with the increase of Ca^2+^, and the SP content decreased at 0 mmol/L < Ca^2+^ ≤ 100 mmol/L, increased sharply when 100 mmol/L < Ca^2+^ ≤ 200 mmol/L, and decreased when Ca^2+^ > 200 mmol/L (Fig. 5-A).

With the extension of culture time, the variation trend and range of SP content in lithophytic bryophytes are different at the same concentration.

When Ca^2+^ = 50 mmol/L, the SP content of *Didymodon constrictus* increased and decreased with the prolongation of culture time, *Eurohypnum leptothallum* showed a downward trend, however the changes of *Hyophila involute* is relatively jumping (Fig. 5-B). When Ca^2+^ = 100 mmol/L, with the extension of culture time, the SP content of *Hyophila involut* and *Didymodon constrictus* increased from 1 to 3 days and decreased after 3 days, *Eurohypnum leptothallum* shows a downward trend as a whole (Fig. 5-C). When Ca^2+^ = 200 mmol/L, with the extension of culture time, the change trend of SP content in *Hyophila involut* and *Didymodon constrictus* was the same before 5 days, which showed that they all decreased from 1 to 2 days, increased from 2 to 3 days, and decreased after 3 days, but *Didymodon constrictus* began to increase after 5 days to 7 days, *Eurohypnum leptothallum* decreased from 1 to 3 days, increased from 3 to 5 days, and increased from 5 to 7 days (Fig. 5-D). When Ca^2+^ ≥ 400 mmol/L, the SP content of the three lithophytic bryophytes showed a downward trend with the extension of culture time, but *Hyophila involut* increased in 3 days -5 days and decreased in 5 days -7 days (Fig. 5-E, F).

### Change characteristics of malondialdehyde (MDA) content

**Fig.6:**
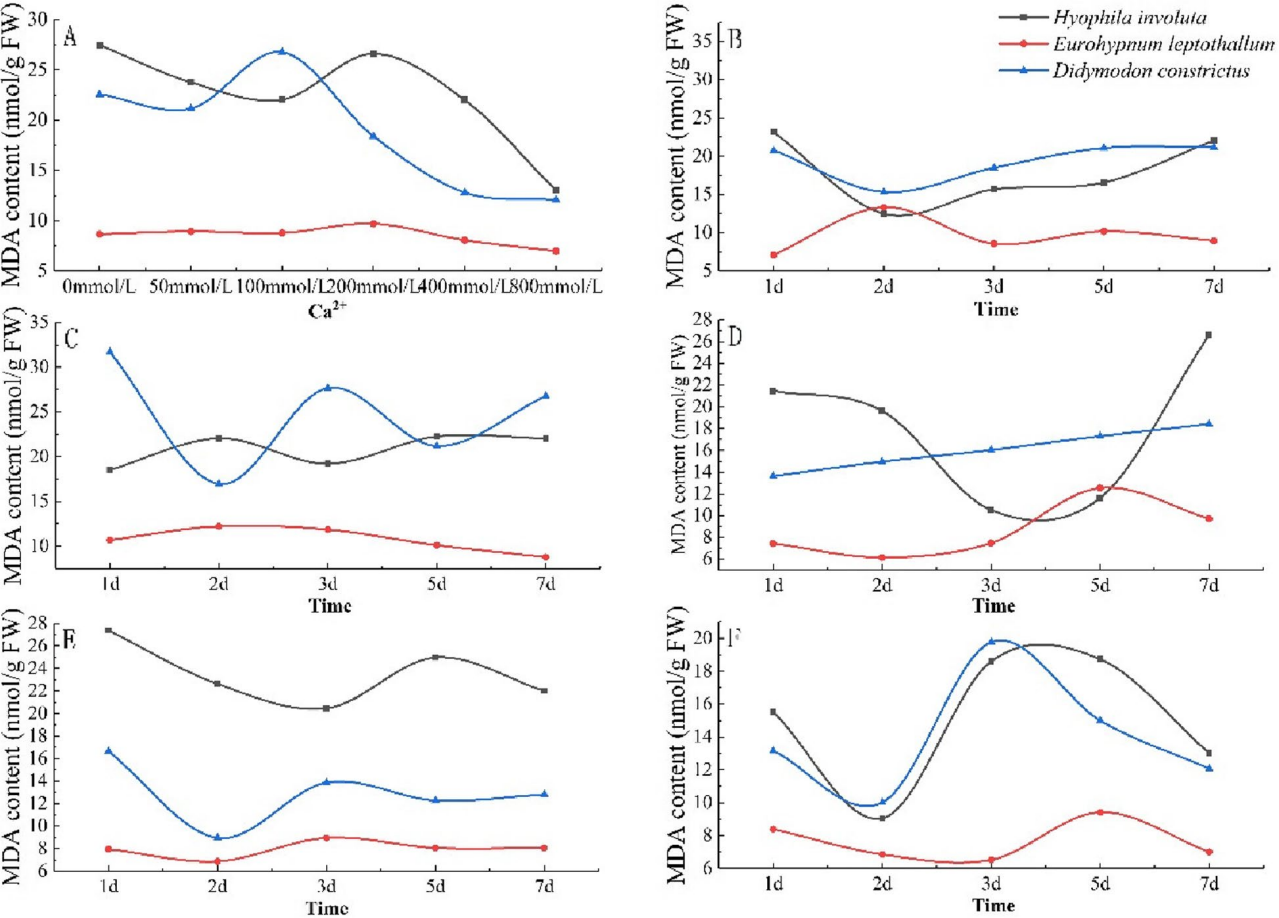
Changes of MDA in lithophytic bryophytes in different calcium environments. **A** The changes of MDA content of three lithophytic bryophytes cultured in different concentrations of Ca^2+^ solution for 7 days. **B** Changes in MDA content of three lithophytic bryophytes after 7 days of culture in Ca^2+^ 50 mmol/L solution. **C** Changes in MDA content of three lithophytic bryophytes after 7 days of culture in Ca^2+^ 100 mmol/L solution. **D** Changes in MDA content of three lithophytic bryophytes after 7 days of culture in Ca^2+^ 200 mmol/L solution. **E** Changes in MDA content of three lithophytic bryophytes after 7 days of culture in Ca^2+^ 400 mmol/L solution. **F** Changes in MDA content of three lithophytic bryophytes after 7 days of culture in Ca^2+^ 800 mmol/L solution

The MDA content of the three lithophytic bryophytes increased with the concentration of Ca^2+^, and the change trend was different among species. Among them, *Hyophila involut* and *Didymodon constrictus* showed a downward upward downward trend, while *Eurohypnum leptothallum* showed an upward downward trend, and its MDA content value was lower than that of *Hyophila involut* and *Didymodon constrictus* at different concentrations(Fig. 6-A). The MDA content of *Didymodon constrictus* decreased when mmol/L 0 ≤ Ca^2+^ ≤ 50 mmol/L, increased when 50 mmol/L < Ca^2+^ ≤ 100 mmol/L, and decreased when Ca^2+^ > 100 mmol/L. MDA content of bryophytes in *Hyophila involut* decreased when mmol/L 0 ≤ Ca^2+^ ≤ 100 mmol/L, increased when 100 mmol/L < Ca^2+^ ≤ 200 mmol/L, and decreased when Ca^2+^ > 200 mmol/L. The MDA content of *Eurohypnum leptothallum* did not change when 0 mmol/L ≤ Ca^2+^ ≤ 100 mmol/L, increased slowly when 100 mmol/L < Ca^2+^ ≤ 200 mmol/L, and decreased when Ca^2+^ > 200 mmol/L, and the MDA content of *Eurohypnum leptothallum* in different concentrations of Ca^2+^ was lower than that of *Hyophila involut* and *Didymodon constrictus*.

When Ca^2+^ = 50 mmol/L, the MDA content of *Hyophila involut* and *Didymodon constrictus* showed the same trend with the extension of culture time, both decreased from 1 to 2 days, and began to rise after 2 days. However, the change trend of *Eurohypnum leptothallum* is opposite to that of the two front mosses, rising from 1 to 2 days and declining after 2 days (Fig. 6-B). When Ca^2+^ = 100 mmol/L, with the extension of time, the MDA content of *Didymodon constrictus* showed a "W" type change trend, while the change range of *Hyophila involut* and *Eurohypnum leptothallum* was not large (Fig. 6-C). When Ca^2+^ = 200 mmol/L, with the extension of culture time, the MDA content of *Hyophila involut* decreased from 1 to 3 days, and increased from 5 to 7 days; Moss rose from 2 to 5 days and declined from 5 to 7 days. *Didymodon constrictus* showed a slow upward trend (Fig. 6-D). When Ca^2+^ = 400 mmol/L, the MDA content of *Hyophila involut* was significantly higher than that of *Didymodon constrictus* and *Eurohypnum leptothallum*, which decreased from 1 to 3 days, increased from 3 to 5 days, and decreased after 5 days. The change trend of *Didymodon constrictus* and *Eurohypnum leptothallum* is the same, which decreases from 1 to 2 days, increases from 2 to 3 days, and decreases after 3 days (Fig. 6-E). When Ca^2+^ = 800 mmol/L, the change trend of *Didymodon constrictus* and *Hyophila involut* is the same, which decreases from 1 to 2 days, increases from 2 to 3 days, and decreases after 3 days. *Eurohypnum leptothallum* decreased from 1 to 3 days, increased from 3 to 5 days, and decreased after 5 days (Fig. 6-F).

### Change characteristics of antioxidant enzyme system

#### Change characteristics of superoxide dismutase (SOD) activity

**Fig. 7:**
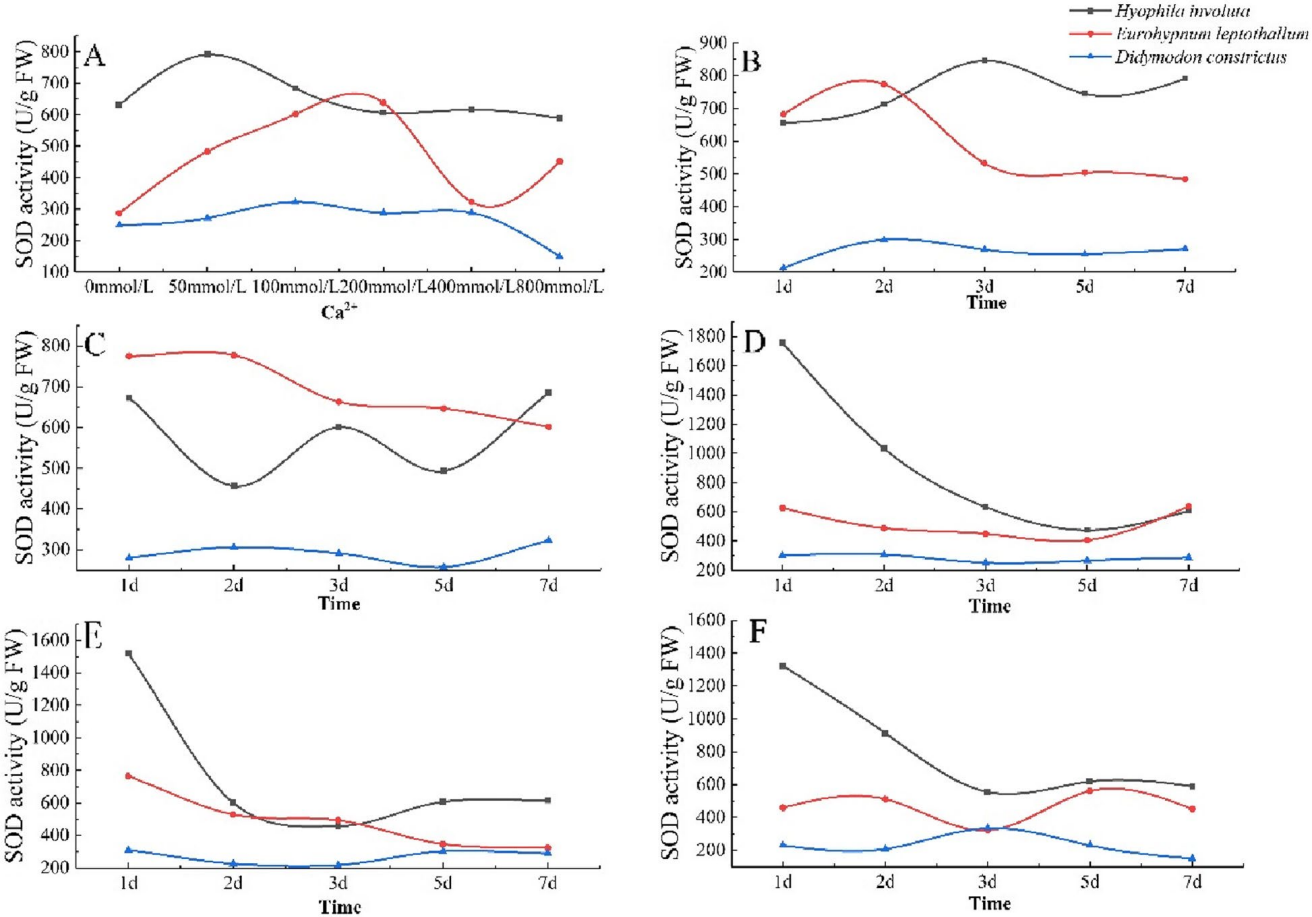
Changes of SOD in lithophytic bryophytes in different calcium environments. **A** The changes of SOD content of three lithophytic bryophytes cultured in different concentrations of Ca^2+^ solution for 7 days. **B** The changes of SOD content of three lithophytic bryophytes after 7 days in Ca^2+^ 50 mmol/L solution. **C** The changes of SOD content of three lithophytic bryophytes after 7 days in Ca^2+^ 100 mmol/L solution. **D** The changes of SOD content of three lithophytic bryophytes after 7 days of culture in Ca^2+^ 200 mmol/L solution. **E** The changes of SOD content of three lithophytic bryophytes after 7 days in Ca^2+^ 400 mmol/L solution. **F** Changes of SOD content of three lithophytic bryophytes after 7 days of culture in Ca^2+^ 800 mmol/L solution

The SOD activity of lithophytic bryophytes showed an overall upward downward trend with the increase of Ca^2+^ concentration, and the SOD activity of *Hyophila involut* was higher than that of *Didymodon constrictus* and *Eurohypnum leptothallum* in different Ca^2+^ concentrations.The SOD activity was the strongest when the Ca^2+^ = 50 mmol/L, 200 mmol/L, and 100 mmol/L of *Hyophila involut*, *Didymodon constrictus* and *Eurohypnum leptothallum*, respectively. When the Ca^2+^ concentration was higher than this concentration, the SOD activity would decrease(Fig. 7-A).

When Ca^2+^ = 50 mmol/L, the SOD activity of lithophytic bryophytes showed an upward downward trend with the extension of culture time. The change trend of SOD activity of *Eurohypnum leptothallum* was the same as that of *Didymodon constrictus*, which increased from 1 to 2 days and decreased after 2 days.The *Hyophila involut* increased from 1 to 3 days and decreased after 3 days (Fig. 7-B). When Ca^2+^ = 100 mmol/L, the SOD activity of *Hyophila involut* showed a "W" trend with the extension of culture time, *Didymodon constrictus* increased from 1 to 2 days, decreased from 2 to 5 days, and increased from 5 to 7 days. *Eurohypnum leptothallum* show a downward trend (Fig. 7-C). When Ca^2+^ = 200 mmol/L, the SOD activity of *Hyophila involut* and *Eurohypnum leptothallum* had the same trend with the extension of culture time, decreased from 1 to 5 days, and increased from 5 to 7 days. There was no significant change in SOD activity of *Didymodon constrictus* (Fig. 7-D). When Ca^2+^ = 400 mmol/L, the SOD activity of the three lithophytic bryophytes decreased as a whole with the extension of culture time, but there were also interspecific differences. Among them, the SOD activity of *Hyophila involut* and *Didymodon constrictus* showed the same trend with the extension of culture time, decreasing from 1 to 3 days and increasing after 3 days (Fig. 7-E).When Ca^2+^ = 800 mmol/L, SOD activity decreased with the extension of culture time, but increased after 3 days. SOD activity of *Hyophila involut* showed a "M" type change trend. The change trend of SOD activity of *Didymodon constrictus* showed an upward downward trend, increased from 1 to 3 days, and decreased after 3 days (Fig. 7-F).

### Characteristics of peroxidase (POD) activity changes

**Fig. 8:**
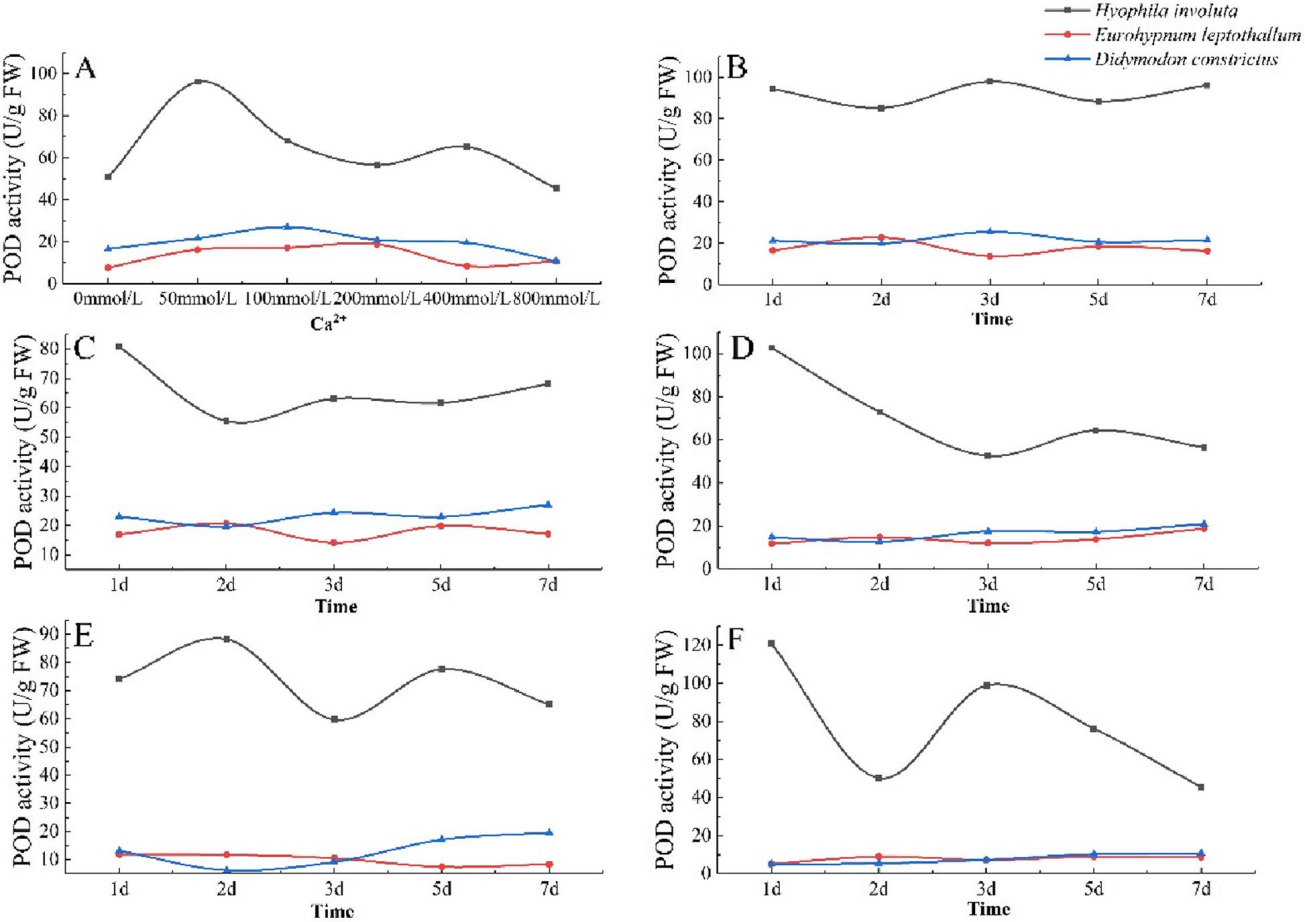
Changes of POD in lithophytic bryophytes in different calcium environments. **A** The changes of POD content of three lithophytic bryophytes cultured in different concentrations of Ca^2+^ solution for 7 days. **B** Changes of POD content of three lithophytic bryophytes after 7 days of cultivation in Ca^2+^ 50 mmol/L solution. **C** Changes of POD content of three lithophytic bryophytes after 7 days of cultivation in Ca^2+^ 100 mmol/L solution. **D** Changes of POD content of three lithophytic bryophytes after 7 days of cultivation in Ca^2+^ 200 mmol/L solution. **E** Changes of POD content of three lithophytic bryophytes after 7 days of cultivation in Ca^2+^ 400 mmol/L solution. **F** Changes in POD content of three lithophytic bryophytes after 7 days of culture in Ca^2+^ 800 mmol/L solution

The POD activity of the three lithophytic bryophytes showed an overall upward downward trend with the increase of Ca^2+^ concentration. The POD activity of *Hyophila involut* was significantly higher than that of *Didymodon constrictus* and *Eurohypnum leptothallum*, and the POD activity of *Didymodon constrictus* and *Eurohypnum leptothallum* was similar. When Ca^2+^ = 50 mmol/L, the POD activity of *Hyophila involut* is the highest. When Ca^2+^ = 200 mmol/L, the POD activity of *Eurohypnum leptothallum* is the highest.When ca2 +  = 100 mmol/L, the POD activity of *Didymodon constrictus* is the highest. When the concentration of Ca^2+^ is greater than this concentration, the POD activity of lithophytic bryophytes decreases (Fig. 8-A).

When Ca^2+^ = 50 mmol/L, the POD activity of the three lithophytic bryophytes was relatively stable and changed slightly with the extension of culture time (Fig. 8-B). When Ca^2+^ = 100 mmol/L, with the extension of culture time, the POD activity of *Hyophila involut* decreased from 1 to 2 days, and increased after 2 days. The POD activity of *Didymodon constrictus* showed a slow upward trend, and the POD activity of *Eurohypnum leptothallum* changed slightly (Fig. 8-C). When Ca^2+^ = 200 mmol/L, the POD activity of *Hyophila involut* showed a downward trend as a whole, increased from 3 to 5 days, and decreased after 5 days. The change trend of POD activity of *Didymodon constrictus* and *Eurohypnum leptothallum* is the same, showing a slow increase (Fig. 8-D). When Ca^2+^ = 400 mmol/L, the change of POD activity of *Hyophila involut* showed a "M" type; *Didymodon constrictus* decreased from 1 to 2 days, and increased after 2 days. The POD activity of *Eurohypnum leptothallum* showed a downward trend with the extension of culture time (Fig. 8-E). When Ca^2+^ = 800 mmol/L, with the extension of culture time, the POD activity of *Hyophila involut* showed a downward trend, decreased from 1 to 2 days, increased from 2 to 3 days, and decreased after 3 days. The POD activity of *Didymodon constrictus* and *Eurohypnum leptothallum* was low, and there was no significant change with the extension of culture time (Fig. 8-F).

### Effects of Osmoregulation Substances on the growth of lithophytic bryophytes

**Fig. 9:**
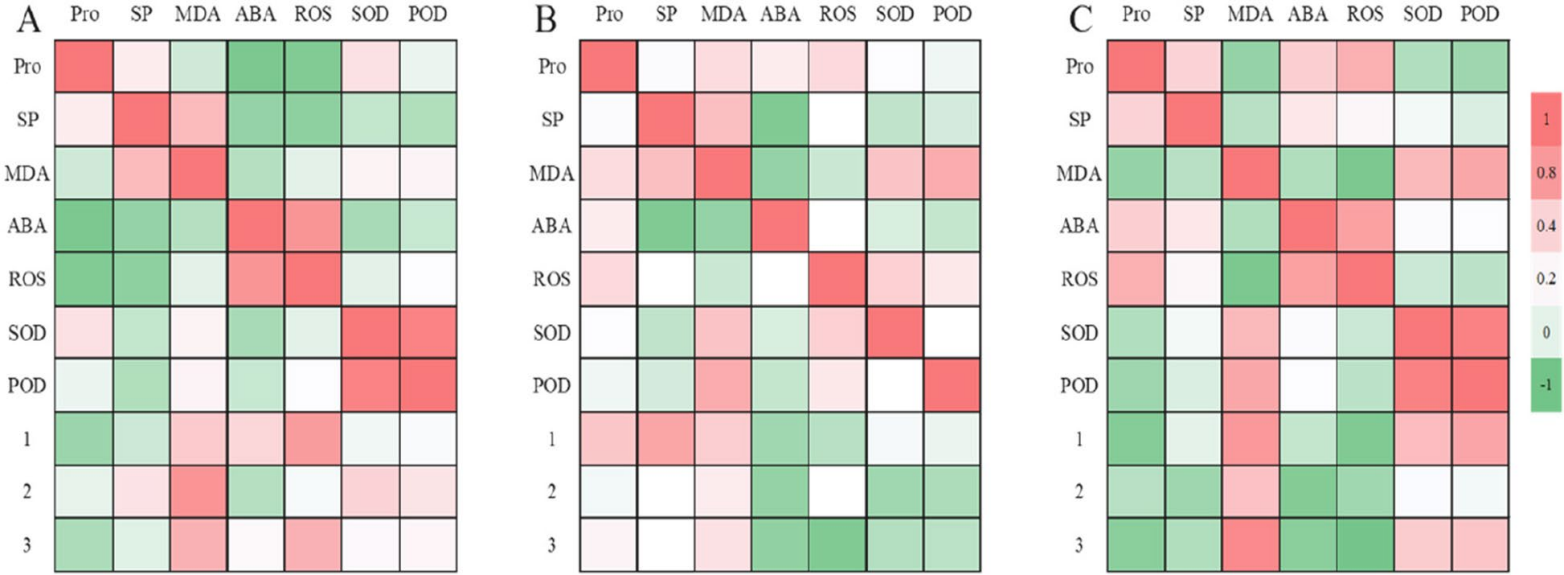
Correlation analysis between the accumulation of osmolyte and plant growth in lithophytic bryophytes under calcium stress. **A** Correlation diagram between the growth of *Hyophila involut* in high calcium environment and o osmolyte. **B** The correlation diagram between the growth volume and osmolyte of *Eurohypnum leptothallum* in high calcium environment. **C** The correlation diagram between the growth and osmolyte of *Didymodon constrictus* in high calcium environment. 1: The growth of lithophytic bryophytes in Ca^2+^ medium in the first month, 2: The growth of lithophytic bryophytes in Ca^2+^ medium in the second month,3: The growth of lithophytic bryophytes in Ca^2+^ medium for 2 months

The growth of *Hyophila involut* was not correlated with SP, SOD and POD as a whole, but positively correlated with MDA (0.50 < *p* < 0.83). ROS was positively correlated with the total growth (*P* = 0.66) and the growth in first month (*p* = 0.78), while Pro and ABA were negatively correlated with total growth and the growth in second month (Fig. 9-A). Pro is negatively correlated with ABA (*p* = -0.82) and ROS (*p* = -0.77). SP was positively correlated with MDA (*p* = 0.60), and negatively correlated with ABA (*p* = -0.63), ROS (*p* = -0.67), SOD and pod. ABA was positively correlated with ROS (*p* = 0.82), and negatively correlated with SOD and POD. There was a very significant positive correlation between SOD activity and POD activity (*p* = 0.94).

The total growth (*p* = 0.94) and the growth in the second month(p = 0.95) of *Eurohypnum leptothallum* were significantly positively correlated with SP (Fig. 9-B).ROS was negatively correlated with the growth in the second month (*p* = -0.87) and total growth (*p* = -0.80), positively correlated with ABA, and positively correlated with Pro, SOD and POD, but the correlation was not significant. SP was significantly negatively correlated with ABA (*p* = -0.80) and ROS (*p* = -0.88). ABA was positively correlated with Pro, but the correlation was not significant, and negatively correlated with MDA (*p* = -0.80) and SP (*p* = -0.65). There was a significant positive relationship between SOD activity and POD activity (*p* = 0.95).

The growth of *Didymodon constrictus* was not correlated with SP as a whole, but negatively correlated with Pro, ABA and ROS (*P* < 0.50), and positively correlated with MDA (0.55 < p > 0.90) (Fig. 9-C). Pro is positively correlated with SP, ABA, ROS (*p* = 0.66), and negatively correlated with MDA (*p* = -0.61), SOD, pod (*p* = -0.55). MDA was negatively correlated with ABA and ROS (*p* = -0.82), and positively correlated with SOD and pod (*P* > 0.61). ABA was positively correlated with ROS (*p* = 0.75). There was a significant positive relationship between SOD activity and POD activity (*p* = 0.94).

### Changes of photosynthetic pigments in lithophytic bryophytes under calcium stress

**Fig. 10:**
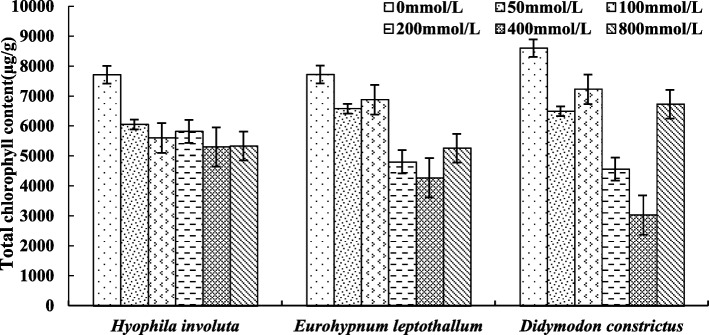
Changes in total chlorophyll of lithophytic bryophytes

The TChl content of the three lithophytic bryophytes decreased with the increase of Ca^2+^ concentration, but there were interspecies differences among the concentrations (Fig. 10). The TChl content of *Hyophila involut* began to decrease when Ca^2+^ > 0 mmol/L, but the difference was not significant at each concentration. The TChl content of *Eurohypnum leptothallum* and *Didymodon constrictus* decreased greatly when Ca^2+^ > 100 mmol/L, which was significantly different from 50 mmol/L and 100 mmol/L, but the TChl content began to rise again when Ca^2+^ = 800 mmol/L. And more than 200 mmol/L and 400 mmol/L TChl content.

**Fig. 11:**
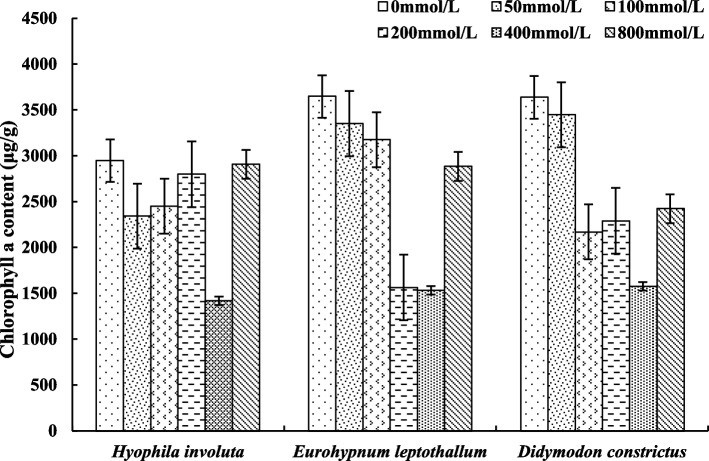
Changes of Chla under calcium stress

The content of Chla in the three lithophytic bryophytes decreased with the increase of Ca^2+^ concentration, but there were interspecific differences in the change law (Fig. 11). The Chla content of the three lithophytic bryophytes decreased when 0 mmol/L < Ca^2+^ ≤ 400 mmol/L, and began to rise when Ca^2+^  = 800 mmol/L. However, at 100 mmol/L < Ca^2+^  ≤ 200 mmol/L, there are different trends. Chla content of *Hyophila involut* increases at 200 mmol/L, while *Eurohypnum leptothallum* and *Didymodon constrictus* decrease.

**Fig. 12:**
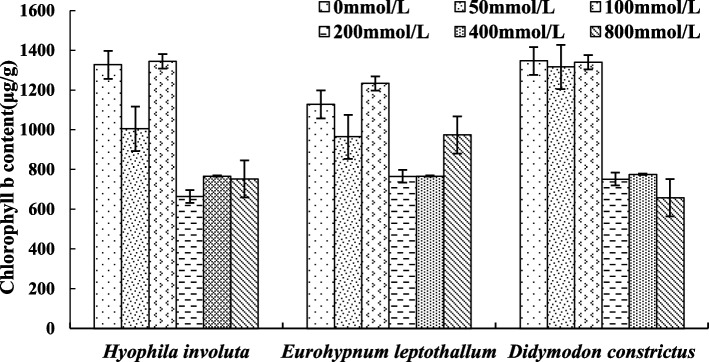
Changes of Chlb content in lithophytic bryophytes under calcium stress

The Chlb content of the three lithophytic bryophytes showed an overall downward trend with the increase of Ca^2+^ concentration, but there were similarities and differences between each species (Fig. 12).

The similar characteristics are as follows: the chlb content of the three lithophytic bryophytes decreases at Ca^2+^ = 50 mmol/L, and increases at 100 mmol/L, which is equivalent to the value of 0 mmol/L, it decreases when Ca^2+^ = 200 mmol/L. The difference is that when Ca^2+^ ≥ 400 mmol/L, the Chlb content of *Eurohypnum leptothallum* is equivalent to 200 mmol/L, and the Chlb content of *Hyophila involut* is greater than 200 mmol/L. And at 800 mmol/L, the amount of *Eurohypnum leptothallum* rose, and exceeded 200 mmol/L and 400 mmol/L, the Chlb content of *Hyophila involut* and *Didymodon constrictus* decreased.

By analyzing the correlation between ABA and chlorophyll of lithophytic bryophytes in different calcium environments (Table 2), it is concluded that ABA and tchl contents are significantly negatively correlated with Chla content, of which *Hyophila involut* is weakly negatively correlated, *Eurohypnum leptothallum* is moderately negatively correlated, and *Didymodon constrictus* is significantly negatively correlated with toothed moss. The correlation between ABA and chlb varies greatly among species. In *Hyophila involut*, ABA and chlb are positively correlated, while in *Eurohypnum leptothallum* and *Didymodon constrictus*, ABA and chlb are moderately negatively correlated. Among the three lithophytic bryophytes, there was a positive correlation between tchl and Chla, but there was an interspecific difference in the correlation with chlb. Among them, the tchl and Chla and chlb of *Hyophila involut* and *Didymodon constrictus* showed a negative correlation, while *Eurohypnum leptothallum* showed a significant positive correlation.

**Table 2:** Correlation between ABA and chlorophyll

		Chlorophyll b content (μg/g)	Total chlorophyll content(μg/g)	Chlorophyll a content (μg/g)
	ABA	0.72	-.842^b^	-0.359
*Hyophila involuta*	chlorophyll b content (μg/g)		-0.597	-0.231
	Total chlorophyll content(μg/g)			0.452
	ABA	-0.591	-.855^b^	-0.64
*Eurohypnum leptothallum*	Chlorophyll b content (μg/g)		.866^b^	.855^b^
	Total chlorophyll content(μg/g)			.917^b^
	ABA	-0.633	-0.722	-.988^a^
*Didymodon constrictus*	Chlorophyll b content (μg/g)		0.669	0.64
	Total chlorophyll content(μg/g)			0.751

^a^ Correlation is significant at the 0.01 level (2-tailed)

^b^ Correlation is significant at the 0.05 level (2-tailed)

## Discussion

### The role of osmolyte in the adaptation of lithophytic bryophytes to a high Ca environment

Environmental stress can cause various adverse effects on plants, such as osmotic stress, ionic toxicity and nutritional imbalance [28], and the accumulation of inorganic and organic solutes involved in osmotic regulation is an important mechanism for plants to adapt to environmental stress [29].The Pro content of *Hyophila involut*, *Eurohypnum leptothallum* and *Didymodon constrictus* were the lowest when the concentration of Ca^2+^ was 200 mmol/L, 50 mmol/L and 50 mmol/L respectively, the SP content was the lowest when the concentration was 100 mmol/L, 100 mmol/L and 50-100 mmol/L, and the MDA content was the lowest when the concentration was 100 mmol/L, 100 mmol/L and 50 mmol/L. It shows that different kinds of lithophytic bryophytes have different calcium adaptability. The optimum growth calcium concentration of *Hyophila involut* is 100 mmol/L-200 mmol/L, the optimum calcium concentration for the growth of *Eurohypnum leptothallum* and *Didymodon constrictus* is 50 mmol/L-100 mmol/L.

Combined with the growth of the three lithophytic bryophytes, they can also grow normally in the culture medium with Ca^2+^ concentration of 200 mmol/L. This is consistent with the research results that the addition of 200 mmol/L CaCl_2_ to the culture medium will promote the growth of *Didymodon tectorus* [30]. In addition, the changes of osmolyte in lithophytic bryophytes with the increase of Ca^2+^ concentration are different from the uniformity of changes of osmolyte in vascular plants under stress [31–33]. The morphological structure and functional traits of organisms are closely related to physiological and ecological processes [34]. Bryophytes are a special group of higher plants that transition from aquatic to terrestrial [35]. They have a special life history, developed gametophytes, and no vascular tissue, and are very sensitive to the environment [36]. They have their own special ways of nutrient absorption and water transmission, thus showing significantly different ecological adaptation characteristics from other higher plants. This may be the reason why bryophytes' physiological changes in response to Ca stress are different from vascular bundle plants.

The calcium tolerance of lithophytic bryophytes is stronger than that of calcicole in karst areas. The optimal calcium concentration of *Eurycorymbus cavaleriei* (50 mmol/L) [12], *Carpinus pubescens* (50 mmol/L) [37], and other non calcicole, such as *Fraxinus mandshurica* (200 mg/kg) [38]. Under most adverse conditions, the accumulation of MDA, SP and Pro is not conducive to plant growth, and with the deepening of stress, the accumulation of these calcicole will increase rapidly [39]. However, the results of this study show that the accumulation of MDA, SP and Pro in lithophytic bryophytes does not increase rapidly with the increase of Ca^2+^ concentration, but shows a trend of first decreasing and then increasing. Under low calcium environment (Ca^2+^ < 200 mmol/L), the changes of Pro, MDA and SP contents of the three lithophytic bryophytes were roughly the same with the extension of culture time. However, in the high calcium environment (Ca^2+^ ≥ 400 mmol/L), there are obvious differences in the changes of osmolyte in the three lithophytic bryophytes. The reason for this phenomenon may be the different adaptive strategies chosen by lithophytic bryophytes in response to high Ca stress. *Eurohypnum leptothallum* and *Didymodon constrictus* showed a "passive response" to high Ca stress, while *Hyophila involut* showed a "slow response" and "lazy response" to changes in Ca^2+^ concentration in the environment.

### Effect of antioxidant enzyme system on adaptation of lithophytic bryophytes to high Ca environment

SOD and POD work together in plants to remove excess ROS and protect the membrane structure, so that plants can tolerate and resist adversity damage [13]. This experiment found that the enzyme system of lithophytic bryophytes behaved differently in different concentrations of Ca^2+^ solutions, and the activities of SOD and POD in different concentrations of Ca^2+^ culture solutions of *Hyophila involut* were higher than those of *Eurohypnum leptothallum* and *Didymodon constrictus*. This result shows that the ability of protective enzyme system in different species to resist stress is different. And the function of protective enzyme system in *Hyophila involut* is stronger than that of *Eurohypnum leptothallum* and *Didymodon constrictus*. In addition, when Ca^2+^ < 200 mmol/L, the POD activity of *Eurohypnum leptothallum* and *Didymodon constrictus* had little change with the extension of culture time, when Ca^2+^ > 400 mmol/L, POD activity is very low. This is inconsistent with the results of adding CaCl_2_ greater than or less than 200 mmol/L to the *Didymodon tectorus* culture medium [30]. The different habitats of the two bryophytes may lead to the different functional properties of their antioxidant enzyme systems.

At the same time, under the culture of different concentrations of Ca^2+^, the SOD activity of *Eurohypnum leptothallum* and *Hyophila involut* varied with the extension of culture time, and it was constantly changing in decline and rise. This is different from the research results that the SOD activity of *Platycarya longipes* increased with the extension of culture time and did not change significantly when it was cultured at different concentrations [40]. The reason for this phenomenon may be that the SOD of lithophytic bryophytes shows a good elastic mechanism in the changing calcium environment, which is different from the antioxidant enzyme system of other plant types in the mechanism of stress.

### Changes in photosynthetic characteristics of lithophytic bryophytes under high Ca environment

The maximum net CO_2_ assimilation rate of bryophytes is widely described as lower than the average level of vascular plants [41–44]. Most bryophytes, especially shade tolerant species, require much less saturated light intensity for photosynthesis than higher plants [45, 46]. Moss belongs to C3 plants [47], and there is a special type of CO_2_ fixation in chloroplasts, namely CO_2_ concentration mechanism. The relationship between dark respiration and CO_2_ exchange is complex, which is different from other higher plants [48, 49]. Through the correlation analysis between chlorophyll and ABA, it was found that ABA of the three lithophytic bryophytes was significantly negatively correlated with Tchl and Chla. It shows that with the increase of ABA accumulation in lithophytic bryophytes, the contents of Tchl and Chla will decrease, which will affect the photosynthetic process of plants. Studies have found that spermatophyte induce stomatal closure and reduce photosynthetic rate through ABA [50]. The morphological characteristics, cell spatial structure and composition of bryophytes are quite different from those of vascular plants. In addition, ABA, as a plant endogenous hormone, is the core hormone for plants to respond to stress [51]. ABA improves the survival probability of plants under salt stress by inhibiting the signal transduction of cytokinins [52, 53]. Then under Ca stress, ABA may activate the enzymatic reaction of chlorophyll degradation, lead to the dissolution and transformation of photosynthetic pigments in cells, reduce their content, reduce the ability of electron transfer, and then affect photosynthesis. ABA may play a stress signal transduction function with other hormones or proteins to promote plants to adapt to high Ca environment, and its exact mechanism needs further study.

In addition, the relationship between the three lithophytic bryophytes Chlb, Chla and TChl varies greatly among species. For example, Chla, Chlb and Tchl of *Eurohypnum leptothallum* are significantly positively correlated, while the contents of Chlb and Tchl are significantly negatively correlated. However, Chla, Chlb, and TChl of *Didymodon constrictus* were all positively correlated. It shows that there are differences in photosynthetic characteristics of lithophytic bryophytes, which may be caused by the differences in morphological characteristics, cell composition and structure, living habits and other differences among species. Marschall found that the chlorophyll concentration of bryophytes living in a shady environment is relatively higher than that of typical heliophilous bryophytes [54]. *Hyophila involut* and *Didymodon constrictus* belong to upright cluster type, while *Eurohypnum leptothallum* belongs to creeping type, the difference of gametophyte morphology. *Hyophila involut* is heliophilous moss, resulting in different osmotic adjustment reaction mechanisms. Plants undergo chlorophyll degradation under leaf senescence, fruit ripening, and biotic and abiotic stress [55]. It is worth noting that Chla in *Eurohypnum leptothallum* decreases, Chlb and Tchl will also decrease, while Chlb decreases, Tchl increases instead. It is suggested that Chlb in *Eurohypnum leptothallum* may be transformed into other photosynthetic pigments, or other photosynthetic pigments may be transformed into some chlorophyll to supplement the content of Tchl.

## Conclusion

Although lithophytic bryophytes can grow on the surface of bare rocks, they can grow in habitats without Ca or in calcium environments with low and medium concentrations (Ca^2+^ ≤ 200 mmol/L), and their tolerance to calcium is stronger than vascular bundle plants. The accumulation of ABA leads to the dissolution and transformation of photosynthetic pigments, decreases their content, reduces the ability of electron transfer, and then affects photosynthesis. However, there may be other photosynthetic pigment synthesis pathways in lithophytic bryophytes, so that the chlorophyll content increases at 800 mmol/L. In addition, the SOD activity and POD activity in lithophytic bryophytes are strong, and their antioxidant enzyme system shows a good elastic mechanism in the changing calcium environment. It is inactive at low concentrations, and it will make a resistance response only when the concentration of Ca^2+^ in the environment exceeds its physiological tolerance limit.

In conclusion, the physiological response of lithophytic bryophytes to high Ca stress is different from vascular bundle plants, and the general stress principle is not applicable to lithophytic bryophytes. Lithophytic bryophytes is "slow" to respond to changes in Ca^2+^ concentration in the environment, showing "passive response" or "lazy response".

## Methods

### Materials

Through previous research, it was found that karst lithophytic bryophytes are rich in species. In this study, the dominant species of lithophytic bryophytes in the karst area, *Didymodon constrictus*, *Eurohypnum leptothallum*, and *Hyophila involuta*, were selected as the research objects. The materials required for the experiment were collected from the Puding Karst Rocky Desertification Ecosystem Observation and Research Station of the Chinese Academy of Sciences.

### Moss culture

In this experiment, the method of liquid culture was used for *Didymodon constrictus*, *Eurohypnum leptothallum*, and *Hyophila involuta*. The method as follows: Clean the soil and other magazines on the surface of the bryophyte. Lay a layer of cleaned sponge (1 cm thick) on the bottom of the culture ware (15 cm*15 cm), lay a layer of cleaned non-woven fabric on the sponge, add water to the culture ware (just before the non-woven fabric), then each treatment group was cultured with moss with a fresh weight of 1.0 g, and the water was changed every three days. The cultivation experiments were all completed in a light incubator, the temperature was set to 18 °C, the light was 2400 Lx, and the duration was 12 h.Cultivated in the laboratory for 2 months to eliminate the background value difference caused by the sampling habitat, and began to do treatment experiments with different concentrations of Ca^2+^.

### Calcium solution culture

According to the calcium content in the underlying soil of lithophytic bryophytes, we set six different calcium concentration, 0 mmol/L, 50 mmol/L 100 mmol/L. 200 mmol/L,400 mmol/L. 800 mmol/L. Prepare Ca^2+^ solution with anhydrous CaCl_2_, and then adjust the pH of the solution to 7 with NaOH (consistent with the average pH of the soil at the sampling site). After hydroponic culture for 2 months, culture ware with the same growth state of bryophytes were selected as Ca^2+^ culture objects. First pour out the water in the culture ware, then spray 30 ml of Ca^2+^ solution into each culture ware, mark the ion concentration and treatment time, and set 7 repetitions for each concentration.

### Measurement methods of bryophyte growth

In order to avoid damage to the growth of bryophytes, the plant height of bryophytes was measured twice, once before the beginning of the experiment and once at the end of the experiment. During each measurement, use sterile paper to absorb the water on the surface of the plant before measuring. The height measurement adopts a scale with an accuracy of mm. Take photos before the experiment, and after treatment for 1 month and 2 months respectively. Plant height measurement: before the experimental treatment, 10 mosses with good growth and consistent height and color were randomly selected from each culture ware, and marked with a thin line of one color. Their initial height H_0_ was measured. During the stress period, their growth changes under different concentration gradients were observed, and the plant height H_1_ was measured again until the end of the stress. The effect of forcing on plant growth height is h = h_1_-h_0_.

### Methods of physiological indexes of bryophytes

We used a sharp blade to collect the green stems and leaves on the upper part of gametophytes of bryophytes, wrap them with tin foil and mark them for classification, then put them into liquid nitrogen for quick freezing for 3 min, and then put them into a low-temperature refrigerator at -80℃ for storage.

- (1) The content of free proline (Pro) was determined by ninhydrin colorimetric method extracted with yellow salicylic acid. Pro was extracted with sulfosalicylic acid (SA). After heating, Pro reacted with acidic ninhydrin solution to produce red color; After extraction with toluene, determine the absorbance at 520 nm. According to the proportion of tissue mass (g): volume of extract (mL): 1:5, homogenate; After that, put it in 95℃ water bath to shake and extract for 10 min; 10000 g, centrifuged at 25℃ for 10 min, take the supernatant, and cool it to be tested. Determine according to the operation procedure in the instructions of the Pro content detection kit.
- (2) Malondialdehyde (MDA) was determined by thiobarbituric acid colorimetry [56]. The condensation of MDA with thiobarbituric acid (TBA) produces red products, with the maximum absorption peak at 532 nm. After color comparison, the content of lipid peroxide in the sample can be estimated. At the same time, determine the absorbance at 600 nm, and calculate the content of MDA by using the difference between the absorbance at 532 nm and 600 nm. According to the proportion of tissue mass (g): volume of extract (mL) 1:5, carry out ice bath homogenization, 8000 g, centrifugation at 4℃ for 10 min, take the supernatant, and put it on ice for testing.Follow the instructions on the MDA content detection kit to conduct the process measurement.
- (3) Coomassie brilliant blue method G-250 was used to determine the content of soluble protein in bryophytes [56]. Prepare Coomassie Brilliant Blue G-250 reagent, weigh 100 mg of Coomassie Brilliant Blue G-250, dissolve it in 50 ml of 95% ethanol, add 100 mL of 85% (m/V) phosphoric acid, and finally fix the volume of distilled water to 1000 mL. Prepare 95% ethanol, 85% phosphoric acid, 1000 μg/mL and 100 μg/mL bovine serum albumin (BSA,OD_280_ = 6.61 at 10 mg/mL generally). Draw standard curves. For protein extraction, accurately weigh 0.50 g of bryophyte, grind the homogenate with 5 mL of distilled water, centrifuge, and reserve the supernatant. To determine the concentration of protein, take 0.1 mL of sample extract, put it into a test tube with a stopper, add 5 mL of Coomassie Brilliant Blue G-250 reagent, fully mix it, place it for 2 min, compare the color at 595 nm, record the absorbance, and check the protein content through the standard curve.
- (4) Superoxide dismutase (SOD) inhibits the reduction of nitroblue tetrazole (NBT) under light to determine the enzyme activity [56]. First, prepare reagents, 50 mmol/L phosphate buffer (pH = 7.8), extraction medium: 50 mmol/L phosphate buffer (pH = 7.8) contains 10 g/L polyvinylpyrrolidone, 130 mmol/L methionine (Met) solution, 750 μg/mL nitrogen blue tetrazole (NBT) solution, 100 μg/mL EDTA-Na_2_ solution, 20 μg/mL riboflavin solution, and all reagents are stored at low temperature and away from light. Secondly, extract the crude enzyme solution. Take 0.5 g of bryophyte and put it into a precooled mortar. Add 2 ml of precooled extraction medium to grind it into a homogenate on an ice bath. Transfer all the homogenate into a centrifuge tube and wash the mortar with the extraction medium to make the final volume of 10 ml. Centrifuge it at 4℃ at 12,000 r/min for 30 min.The supernatant is the crude extract of SOD, which is stored at low temperature. Finally, take five 25 ml glass tubes for enzyme activity test, three of which are measuring tubes and two are control tubes. Add the prepared reagents respectively. After mixing, place one control tube in a dark place. The other tubes react under 4000 lx sunlight for 20 min at 25℃. After the reaction, put them in a dark place to stop the reaction. Use a non illuminated control tube as a blank reference to adjust zero, and measure the absorbance of other tubes at 560 nm.
- (5) The peroxidase (POD) activity was determined by guaiacol colorimetry [56].First, prepare 0.1 mol/L phosphoric acid buffer solution (pH = 6.0), reaction mixture: take 50 mL of 0.1 mol/L phosphoric acid buffer solution reagent (pH = 6.0) in a beaker, add 28 μg of guaiacol, heat and stir on a magnetic mixer until the pre traumatic lignol is dissolved, add 19μL 30% H_2_O_2_ after the solution cools, mix and stir, mix well, and store at low temperature. Next, extract the crude enzyme solution, weigh 1 g of bryophyte, put it into a cold mortar, add 5 mL of pH phosphoric acid buffer solution, grind it into a homogenate, transfer it into a 100 mL volumetric flask, wash the residue into the volumetric flask with phosphoric acid buffer solution, and then dissolve it. After mixing, take a certain amount and put it into a centrifuge tube, centrifuge at 4000r/min at 4℃ for 10 min, collect the supernatant, and put it in a 4℃ refrigerator for standby. Finally, determine the enzyme activity, take two cuvettes with a light diameter of 1 cm, and add 3 mL of reaction mixture and 1 mL of phosphate buffer into one cuvette as a blank control. Add 3 mL of reaction mixed solution and 1 mL of crude enzyme solution into the other one. When using the stopwatch immediately, measure the change of absorbance (A_470_) at 470 nm, and read the value every 1 min.
- (6) Detection method of ABA. Weigh about 0.1 g of bryophyte, add 1 mL of precooled reagent I (methanol: water: acetic acid = 80:20:1), extract overnight at 4℃, centrifugate 8000 g for 10 min, and extract the residue with 0.5 mL of reagent I (methanol: water: acetic acid = 80:20:1) for 2 h. After centrifugation, take out the supernatant, combine the supernatant extracted twice, blow nitrogen at 40℃ until there is no organic phase, add 0.5 mL of reagent II (petroleum ether) to extract and decolorize for three times, and discard the upper ether phase, Add reagent III (saturated citric acid aqueous solution) to adjust the PH to 2.8, extract the organic phase three times with reagent IV (ethyl acetate).Blow the nitrogen until it is dry. Add 0.5 mL reagent V (methanol) to dissolve it by vortex vibration. After filtering with a needle filter, use high-performance liquid chromatography (RIGOL L3000) to detect.
- (7) Determination of chlorophyll: cut the gametophytes of bryophytes, remove impurities, dry the surface water, cut and mix evenly. Weigh about 0.20 g, make five repetitions, respectively put them into a mortar, add a small amount of quartz sand, calcium carbonate and 2 ~ 3 mL of 95% ethanol, grind them into a homogenate, add 10 mL of ethanol, continue to grind until the tissue turns white, stand for 3 ~ 5 min, filter them into a 25 mL brown volumetric flask, wash them with a small amount of ethanol several times until there is no green in the filter paper and residue, and finally fix the volume with 95% ethanol. Colorimetry was used to determine the pigment, with 95% ethanol as the blank, and the absorbance was measured at 665 nm, 649 nm [57].The contents of chlorophyll, chlorophyll a and chlorophyll b were calculated by method Arnon.

### Statistics analysis

The data were statistically analyzed by Excel 2016, SPSS16.0 and other software, and plotted by Origin8.5 software. The data of MDA, SP, Pro, POD, SOD and ABA of karst lithophytic bryophytes cultured at different Ca^2+^ concentrations were tested by one-way ANOVA(α = 0.05).If the variance is homogeneous, the least significant difference (LSD) method is used for multiple significant comparisons.If the variance is not homogeneous, use Tamhanes T2 method for multiple comparisons, and the significance level is(α = 0.05).Pearson correlation analysis method was used to analyze the correlation between the growth of stony bryophytes and the content of osmoregulation substances, antioxidant enzymes and abscisic acid under different Ca^2+^ concentration. The significance level was set as α = 0.05.The data are expressed as mean ± standard deviation.

## Data Availability

The datasets used and/or analysed during the current study available from the corresponding author on reasonable request.

## Abbreviations

Ca: Calcium
Ca^2+^: Calcium ion
MDA: Malondialdehyde
SS: Soluble sugar
ABA: Abscisic acid
SOD: Superoxide dismutase
CAT: Catalase
POD: Peroxidase
ROS: Reactive oxygen species
TChl: Total chlorophyll
Chl a: Chlorophyll a
Chl b: Chlorophyll b
